# Navigation Training for Persons With Visual Disability Through Multisensory Assistive Technology: Mixed Methods Experimental Study

**DOI:** 10.2196/55776

**Published:** 2024-11-18

**Authors:** Fabiana Sofia Ricci, Lorenzo Liguori, Eduardo Palermo, John-Ross Rizzo, Maurizio Porfiri

**Affiliations:** 1 Department of Biomedical Engineering New York University Tandon School of Engineering Brooklyn, New York, NY United States; 2 Center for Urban Science and Progress New York University Tandon School of Engineering Brooklyn, New York, NY United States; 3 Department of Mechanical and Aerospace Engineering Sapienza University of Rome Rome Italy; 4 Department of Mechanical and Aerospace Engineering New York University Tandon School of Engineering Brooklyn, New York, NY United States; 5 Department of Rehabilitation Medicine New York University Langone Health New York, NY United States; 6 Department of Neurology New York University Langone Health New York, NY United States

**Keywords:** assistive technology, human-computer interaction, multisensory feedback, virtual reality, visual impairment, haptic

## Abstract

**Background:**

Visual disability is a growing problem for many middle-aged and older adults. Conventional mobility aids, such as white canes and guide dogs, have notable limitations that have led to increasing interest in electronic travel aids (ETAs). Despite remarkable progress, current ETAs lack empirical evidence and realistic testing environments and often focus on the substitution or augmentation of a single sense.

**Objective:**

This study aims to (1) establish a novel virtual reality (VR) environment to test the efficacy of ETAs in complex urban environments for a simulated visual impairment (VI) and (2) evaluate the impact of haptic and audio feedback, individually and combined, on navigation performance, movement behavior, and perception. Through this study, we aim to address gaps to advance the pragmatic development of assistive technologies (ATs) for persons with VI.

**Methods:**

The VR platform was designed to resemble a subway station environment with the most common challenges faced by persons with VI during navigation. This environment was used to test our multisensory, AT-integrated VR platform among 72 healthy participants performing an obstacle avoidance task while experiencing symptoms of VI. Each participant performed the task 4 times: once with haptic feedback, once with audio feedback, once with both feedback types, and once without any feedback. Data analysis encompassed metrics such as completion time, head and body orientation, and trajectory length and smoothness. To evaluate the effectiveness and interaction of the 2 feedback modalities, we conducted a 2-way repeated measures ANOVA on continuous metrics and a Scheirer-Ray-Hare test on discrete ones. We also conducted a descriptive statistical analysis of participants’ answers to a questionnaire, assessing their experience and preference for feedback modalities.

**Results:**

Results from our study showed that haptic feedback significantly reduced collisions (*P*=.05) and the variability of the pitch angle of the head (*P*=.02). Audio feedback improved trajectory smoothness (*P*=.006) and mitigated the increase in the trajectory length from haptic feedback alone (*P*=.04). Participants reported a high level of engagement during the experiment (52/72, 72%) and found it interesting (42/72, 58%). However, when it came to feedback preferences, less than half of the participants (29/72, 40%) favored combined feedback modalities. This indicates that a majority preferred dedicated single modalities over combined ones.

**Conclusions:**

AT is crucial for individuals with VI; however, it often lacks user-centered design principles. Research should prioritize consumer-oriented methodologies, testing devices in a staged manner with progression toward more realistic, ecologically valid settings to ensure safety. Our multisensory, AT-integrated VR system takes a holistic approach, offering a first step toward enhancing users’ spatial awareness, promoting safer mobility, and holds potential for applications in medical treatment, training, and rehabilitation. Technological advancements can further refine such devices, significantly improving independence and quality of life for those with VI.

## Introduction

### Background

Visual impairment (VI) affects a considerable proportion of middle-aged and older adults [1]. In the United States alone, approximately 12 million people aged ≥40 years experience VI, with about a million of them experiencing blindness [2]. Globally, the statistics are similar, with reports from the World Health Organization indicating that there are ≥2.2 billion people with eye and vision problems [3]. Not only is VI an important contribution to mobility disability, it is also associated with increased risks of stroke, arthritis, diabetes, and cancer [4-6]. VI is also significantly associated with decreased life satisfaction, unemployment, and social isolation, which may lead to depression and increased risk of suicidal behavior [7-9]. Considerable economic costs are also associated with VI due to productivity losses, costs to the health system to provide accessible eye care, and other financial implications of vision loss and its comorbid conditions [10,11].

The autonomy of persons with VI is often jeopardized for the many everyday tasks they need to attend to, including travelling unknown environments. One of the chief challenges to achieving independence for persons with VI is associated with safe, independent, and efficient navigation, particularly in unfamiliar locations [12-14]. Conventional navigation aids include white canes and guide dogs [15]. Although these aids provide valuable mobility support, they bear important limitations that preclude their widespread adoption. In fact, only an estimated 2% to 8% of persons with VI use white canes or guide dogs in the United States [16,17]. The white cane is light, portable, and easily replaceable, but it can only detect objects through physical contact. It is unable to provide any information about sublevel pits or holes, uneven terrain, and obstacles that are not in the range of the stick. Likewise, it is difficult to use for detecting moving objects, such as cars and people [18,19]. Guide dogs may help with more security in new and unfamiliar areas and can improve the safety of their owners. However, guide dogs are expensive, their training period is long, and they are only viable for about 7 years [18,19]. In the last 20 years, several studies have focused on assistive devices to foster independence and facilitate navigation of persons with VI in indoor and outdoor environments. These technologies, known as electronic travel aids (ETAs), are devices that collect environmental information using 1 or more sensors and transmit such information to the user through touch and sound [20]. The state of the art offers a wide range of ETAs that incorporate functions for obstacle avoidance or r route selection [21-23].

Development of ETAs with regard to production and commercialization is still hindered by 2 main factors [24].

The first factor is the lack of empirical evidence about the extent to which such devices detect obstacles and improve performance in mobility tasks [25]. In fact, most systems developed for persons with VI have concentrated on addressing the deficit of sight through the enhancement of a singular sensory input. Often, the emphasis has been on substituting or augmenting visual information through technologies that cater to touch or sound [26-28]. While these approaches to sensory substitution have shown promising outcomes, they may miss out on the broader advantages of combining multiple senses. Relying on a single sensory modality could limit the overall appraisal of the environment for individuals with VI [29]. A multisensory approach could offer a more nuanced and complete perception of surroundings, paving the way for more effective solutions for persons with VI [30,31].

Second, the state of the art on ETA testing has relied on artificial or noncontrolled settings that limit one’s ability to assess the value of any particular approach before field deployment [32-35]. In particular, users are guided through these setups using game pads or joysticks, which may inadequately emulate the unpredictable challenges encountered in daily life by persons with VI [36]. Experimental validation in these less-than-realistic environments with limited ecological validity might result in an inaccurate estimation of the system’s effectiveness. Real-world conditions introduce a multitude of variables and complexities that are challenging to replicate artificially, emphasizing the need for more comprehensive testing strategies that better reflect the dynamic nature of everyday scenarios.

In this study, we propose an assistive technology (AT) combining haptic and audio cues to provide comprehensive obstacle avoidance assistance. The haptic feedback was delivered through an improved version of the wearable system previously developed by our group [37-39], consisting of a belt equipped with an array of actuators positioned around the user’s abdomen. This tactile interface served as an intuitive guide, conveying real-time information about the proximity of obstacles in the user’s surroundings. The proposed ETA features an audio feedback component that uses beep sounds to alert users to potential obstacles.

We developed a virtual reality (VR) framework to explore the effectiveness of the multisensory AT on healthy participants, before field deployment on persons with VI. VR provides a versatile platform for seamlessly incorporating various haptic feedback modalities and enhancing them with complementary audio effects, thereby facilitating navigation within virtual environments [40,41]. The precision of VR allows for the accurate simulation of diverse, and even rare, forms of eye pathologies [42-44]. The ability to simulate VI has broad applications across science, engineering, and medicine. For example, effective VI simulations could enhance public understanding of VIs, potentially aiding in early disease diagnosis [45-48].

### Study Overview

Our study involved the creation of a realistic and dynamic subway station environment, where 72 healthy participants performed a virtual obstacle avoidance task while experiencing simulated VI. The experiment comprised 4 conditions: haptic feedback only, audio feedback only, both haptic and audio feedback, and no feedback. For each condition, we gathered data on participants’ navigation performance, including time to complete the task, number of collisions, trajectory length, and smoothness, as well as their movement behavior, encompassing head and body orientation. Through a multifaceted comparison of participants’ movement behavior and navigation performance across conditions, we sought to evaluate the role of haptic and audio feedback, both individually and in combination, on users’ mobility and behavior. We envision this platform as a robust and easily customizable tool for investigating diverse feedback modalities, contributing to a deeper understanding of the needs of individuals with VI, and fostering continuous advancements in the design and development of ATs.

## Methods

### VR Platform

#### Design of the Environment

We built a VR platform to assess the effect of different types of feedback modalities and their combination on users’ behavior and navigation performance. VR constitutes an ideal framework to test different conditions in highly realistic and dynamic scenarios [49-51]. We designed a multisensory, AT-integrated VR system comprising audio feedback implemented in VR and a haptic feedback device interfaced with the virtual environment. We conceived an obstacle avoidance task to assess the ability of the 2 feedback modalities (individually or together) to enhance the mobility of persons with VI.

**Figure 1:**
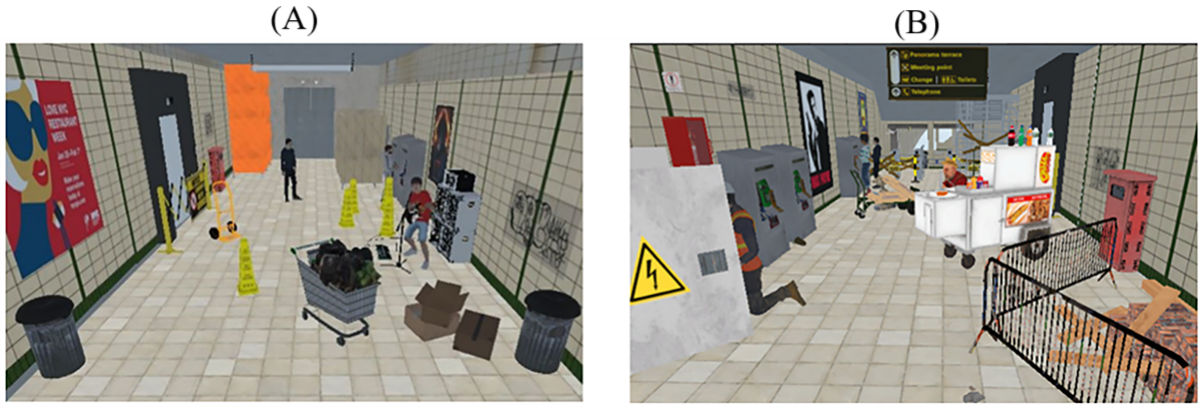
Example of the virtual reality environments implemented in this study: (A) the first floor and (B) second floor of a subway station. We simulated an elevator ride from the first floor to the second floor of the subway station environment.

The application was built and run on a Lenovo Legion 5 15IMH05H gaming laptop. To optimize the gaming stream and ensure the immersiveness of the application, we used a TP-Link Archer GX90 AX6600 Tri-Band Wi-Fi 6 Gaming Router. The Unity game engine (version 2019.4.9f1) was used to develop a VR application for the Meta Quest 2 headset and Touch controllers. Users navigated the virtual environment by physically walking in a first-person perspective. In VR, we designed 2 floors of a subway station whose size matched the dimensions of the physical environment where the experiment took place. The 2 environments included common obstacles and hazards that may be encountered while walking in a subway station, such as broken elevators, construction sites, working tools, garbage, scaffoldings, signage furnishing, and turnstiles (Figure 1). A food vendor, a street musician, and other pedestrians were included to increase engagement and dynamism of the overall environment (Figure 1). We also simulated an elevator ride from the first floor to the second floor of the virtual subway station (Multimedia Appendix 1).

To create a realistic VR experience, sound effects related to a subway station environment, including those of animated pedestrians, were added. As shown by prior studies, integrating sounds related to the visual content enhances the sense of presence of participants in a virtual environment [52,53]. To integrate realistic audio effects in the VR application, we used FMOD, an end-to-end solution for sound that integrates seamlessly with Unity. It simplifies the process of creating sound behaviors, with a comprehensive set of features that allows one to quickly and easily build adaptive audio.

#### VI Simulation

**Figure 2:**
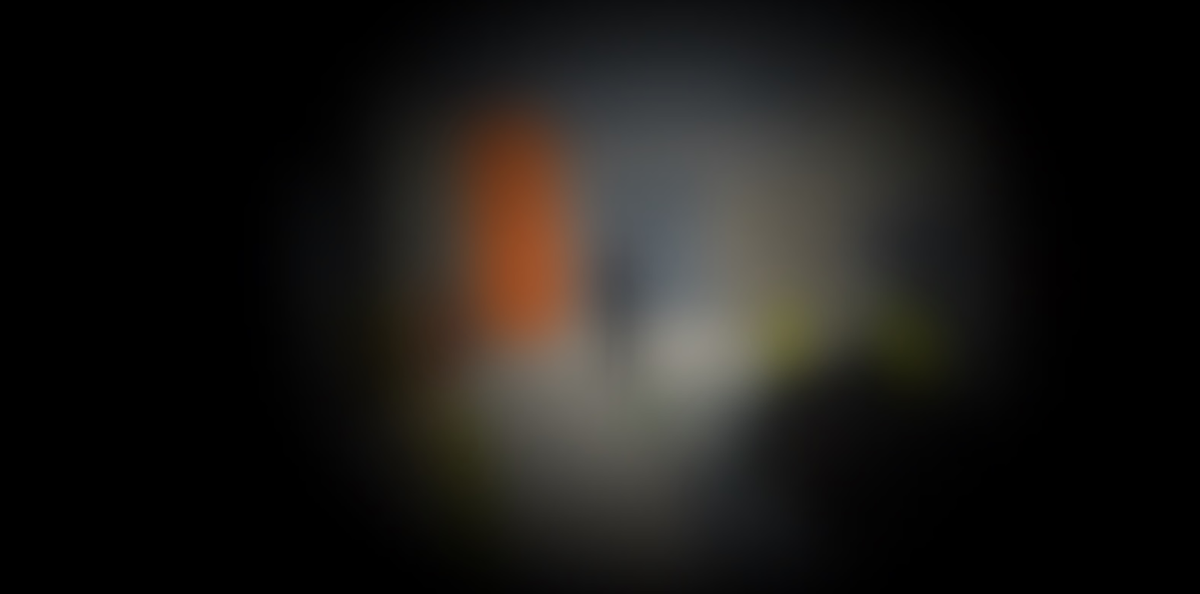
Effects on vision due to a visual impairment simulated in virtual reality: peripheral vision loss, reduced contrast sensitivity, altered color perception, and glare.

In VR, we simulated different aspects of VI, including peripheral vision loss, reduced contrast sensitivity, altered color perception, and glare [54], as shown in Figure 2 (refer to Multimedia Appendix 2 for more details). Impairment severity was based on the extent of peripheral vision loss and the intensity of the simulated symptoms. Our simulation of peripheral vision loss specifically targeted the severe stage of glaucoma, a prevalent cause of VI among adults in the United States, which is known for its substantial impact on mobility [55]. This progressive reduction of the peripheral visual field in glaucoma impedes the clear identification of objects, which is crucial for obtaining wide-field information about the environment [56,57]. Realistic simulation of such symptoms was accomplished by combining postprocessing effects and C# scripts coded in Unity. Specifically, we combined rendering and graphic tools provided by Unity, such as shader and culling mask. A shader is a mini-program that provides a flexible way of dynamically tweaking the appearance of any components in the scene (such as models and lights). A culling mask is a camera’s property that allows one to selectively render objects in the scene. We used a Gaussian blur shader to reproduce the symptoms of glare and blurriness and a culling mask to create the visual effects of peripheral vision loss, reduced contrast sensitivity, and altered color perception.

To ensure the realism and accuracy of our simulations, we sought the expertise of 2 professionals familiar with low-vision conditions. Specifically, a certified orientation and mobility specialist (also a certified low-vision therapist) with ≥30 years of experience in the field and the chief research officer at an American nonprofit organization dedicated to vision rehabilitation and advocacy for the blind, who is also a research professor of ophthalmology at New York University Grossman School of Medicine, provided their expertise.

### Multisensory, AT-Integrated VR System

#### Obstacle Detection

Obstacle detection was implemented using the *UnityEngine.PhysicsModule*. Specifically, the *Spherecast* function was used to project a sphere of a given radius into the scene. The function returns a true Boolean value when an object in the virtual environment is hit by the sphere, and it provides information about the distance between the projection point and the object.

#### Haptic Feedback

**Figure 3:**
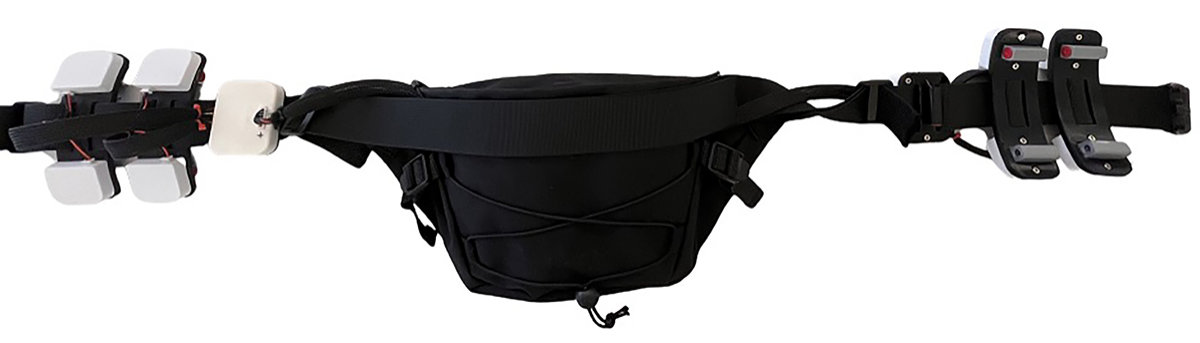
Picture of the new prototype of the haptic feedback device tested in this study.

The haptic feedback was provided by a wearable device in the form of a belt that improves on our team’s previous effort [37-39]. The belt was equipped with 10 cylindrical eccentric rotating mass actuators (Precision Microdrives Ltd, model number 307-103) with a diameter of 9 mm and a length of 25 mm. We opted for this type of actuator as it is widely available, simple to use, and inexpensive. The actuators were arranged on 6 distinct modular units that could be added or removed easily based on users’ preference, ability, and experience with the device (Figure 3). The units were designed in SolidWorks (version 2019) and 3D printed on a Bambu Lab X1C. Precisely, the 4 central modules had 2 actuators each disposed horizontally and separated by a vertical distance of 85 mm. In these central modules, each actuator was enclosed in a parallelepipedal housing of dimensions 35 mm × 42 mm × 10 mm. The housing was made of polylactic acid. To minimize the vibrations inside the modules, each actuator was connected through springs to a flexible element of thermoplastic polyurethane. The 2 modules at the ends of the belt each had a single actuator positioned vertically in the center. In these lateral modules, each actuator was enclosed in a parallelepipedal housing of dimensions 45 mm × 60 mm × 12 mm.

Once assembled, the modules were evenly inserted on the 2 straps of a commercial waist bag, which was secured above the user’s hips through a buckle. Inside the waist bag, we placed all the electronic components needed to control and power the belt, namely, a custom printed circuit board, an EPS32 microcontroller (Espressif Systems), and a Krisdonia 50,000 mAh power bank. The function of the actuators on the belt was to provide environmental information through vibration feedback on the users’ abdomen. Specifically, the vibration indicated the presence and location of obstacles near the user in the virtual environment. The amplitude and frequency of the vibration were programmed to vary on 3 levels based on the distance from the obstacles; information about the position and location of closer obstacles was conveyed through higher amplitude and frequency. The belt was connected to the laptop via Wi-Fi using the EPS32 microcontroller. The interface between the belt and the VR environment was enabled through a server or client transmission control protocol established in a C# script.

The user’s field of view in VR, characterized by a horizontal span of 89° and a vertical span of 93° (per the Meta Oculus Quest 2 specifications), was discretized into a grid comprising 10 sectors. This grid layout closely mirrored the configuration of actuators on the haptic feedback belt. Each sector was then associated with a virtual sphere projected from the user’s body. The 10 resulting spheres were positioned to align with the 10 field of view sectors. Anytime an obstacle fell into a sector, it was detected by a specific sphere, and information to activate the actuators was sent through the transmission control protocol to the EPS32 microcontroller. The latter used pulse width modulation to control a metal-oxide-semiconductor field-effect transistor driver (Texas Instruments) placed in the printed circuit board, which fed the actuators. The maximum hit distance of the spheres was set to 2.5 m based on pilot testing of the haptic feedback system. This value determined the range of action of the belt. The frequency of vibration was regulated on 3 levels based on the distance of the object from the user in VR by means of a C++ code.

#### Audio Feedback

The audio feedback was provided through the VR headset, and it consisted of a beep sound added to the VR application using an FMOD sound effect engine. Similar to haptic feedback, audio feedback serves the purpose of alerting users of the presence of obstacles in their surroundings via a beep sound. The sound was played at increasingly short intervals as the user approached an obstacle. The VR device was connected to the laptop via Wi-Fi using the Oculus application and Quest Link.

Obstacle detection through audio feedback was again implemented in a C# script using the Spherecast function. However, in this case, only 1 sphere was designed to be projected from the user’s head in the virtual environment. Anytime an object was in the direction the user was facing, it was detected by the sphere and a beep sound was emitted by the VR headset to alert the user about the presence of an obstacle. Similar to the haptic feedback, the maximum hit distance of the sphere was set to 2.5 m. The rationale behind this audio feedback design was to enhance users’ residual vision while exploring the environment with their head movement via simple and intuitive audio feedback.

Moving forward, future implementations could explore additional sensory cues to further enrich the user experience in virtual environments. For example, synchronized footstep sounds tailored to users’ movements have been shown to significantly elevate perceived presence in the virtual environment. This heightened presence fosters greater awareness of one’s gait and posture, resulting in more authentic interactions and enhanced movement control [58]. The efficacy of echo-acoustic cues in navigating virtual environments has also been previously assessed [59]; not only could these cues improve collision avoidance and navigation efficiency, but they may also enhance the perception and evaluation of different routes after training.

### Experimental Methods

#### Participants

A total of 72 healthy participants with a mean age of 25.93 (SD 4.48) years were recruited from New York University Tandon School of Engineering. Of these 72 participants, 26 (36%) self-identified as women and 46 (64%) as men. To reduce the risk of injury or discomfort associated with the use of a VR device, we excluded people who were pregnant; older adults; had preexisting binocular vision abnormalities or psychiatric disorders; had a heart condition, seizures, or other serious medical conditions; and used medical devices. We opted for self-reported visual acuity to exclude persons with preexisting binocular vision abnormalities, as conducting objective screenings for all participants would have required additional resources, including time and personnel. Given the nature of our research and the characteristics of our target population, we felt self-reporting was a practical and feasible approach, allowing us to efficiently gather relevant data without significantly extending the duration of participant recruitment and data collection. Participants with normal or normal corrected vision were included in the study.

#### Procedure

**Figure 4:**
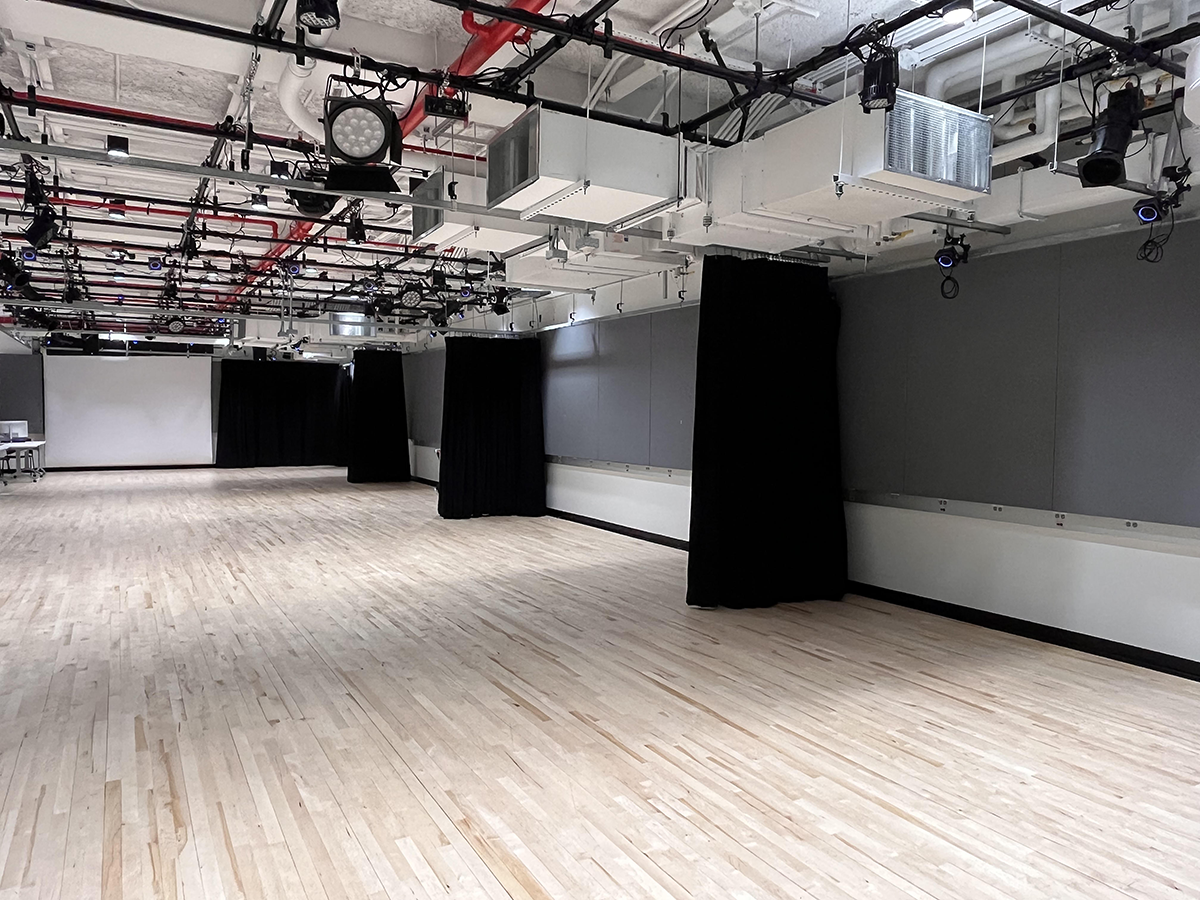
Multipurpose production space used to conduct the experiments.

The experimental study took place in a multipurpose production space at New York University’s Media Commons, consisting of 4 bays, each of which was 6 m long and 2 m wide with a total area of 178 m^2^. Other than 4 curtains positioned along the sidewall of the bays, the environment was free from obstructions. Thus, participants were able to walk freely during the experiment (Figure 4).

There was no training provided for using the haptic feedback device or the VR platform; participants completed the experiment in a single session.

Participants performed an obstacle avoidance task on the 2 floors of the virtual subway station environment while experiencing the most common symptoms and signs of a VI. Specifically, participants were asked to physically walk from a starting point until they reached a virtual elevator and then turn 180° and walk back until they reach the train platform. To help participants understand that they had reached the final destination, arrival was signaled through the sound of a turnstile opening and the animation of a train passing by. Immediately after the completion of each condition, participants were asked to fill out a questionnaire concerning their overall experience and the 2 types of feedback (refer to the Questionnaire subsection).

During the experiment, the belt and the VR headset alerted users about the presence of obstacles in the surrounding environment through vibration feedback on the abdomen and audio feedback, respectively, to minimize the possibility of a collision. The right Oculus Touch controller vibrated any time a user hit an obstacle in the virtual environment to reproduce the sensation of touching an object. The left Oculus Touch controller was attached to the haptic feedback device vertically to track the position of the users during the experiment (refer to the Data Collection subsection). The experiment was aimed at realistically recreating a path from the entrance of a subway station to the train platform, with a maximum duration of 30 minutes to prevent distress associated with extended VR sessions [60].

#### Conditions and Research Questions

A total of 4 experimental conditions were tested to elucidate the individual and combined effects of haptic and audio feedback on movement behavior, navigation performance, and self-reported ratings. Each participant performed the task in 4 different conditions: no feedback, haptic feedback only, audio feedback only, and both feedbacks. Apart from the type of feedback provided, all conditions were identically structured. Each participant was assigned to only 1 (4%) of the 24 possible combinations for the following purposes: (1) preventing fatigue from potentially diminishing the impact of the feedback on users’ performance in the later stages of the experiment and (2) mitigating biases related to increased familiarity with the devices. During the obstacle avoidance task, data on the navigation performance (task completion time, number of collisions, and trajectory) and movement behavior (head and body orientation) of the participants were collected (refer to Data Collection subsection).

This study aimed to answer the following research questions (RQs) based on the collected data:

- RQ1. How did individual and synergistic use of the 2 types of feedback affect the navigation performance of participants across experimental conditions?
- RQ2. How did individual and synergistic use of the 2 types of feedback affect the movement behavior of participants across experimental conditions?
- RQ3. How did participants perceive the individual and synergistic use of the 2 types of feedback across experimental conditions?

### Data Collection

#### Metrics

During each experiment, we collected the following metrics: number of collisions, completion time, head orientation, and body position and orientation. To save these metrics, we used 2 C# scripts. The first script was used to start and reset a stopwatch at the beginning of each experiment and to collect the following data: (1) head orientation (Euler angles) from the VR headset, (2) body position from the user’s body in VR, and (3) body orientation from the left Oculus Touch controller. Specifically, to collect data on users’ body position, we provided the player with a CapsuleCollider and a RigidBody component. The former is an invisible capsule-shaped primitive that represents the user’s body in VR, while the latter provides the user’s body with physics properties. These 2 components moved in the virtual environment according to the movement of the user in the real environment. The left Oculus Touch controller was secured vertically on the belt by means of an element 3D-printed in carbon fiber reinforced polylactic acid and used for collecting users’ body orientation. The game object representing the left Oculus Touch controller in VR moved in the virtual environment according to the movement of the physical controller in the real environment.

The second script was used to simulate the collision with obstacles and to alert the user through a vibration provided by the right Oculus Touch controller. To enable the vibration of the controller, each virtual object was provided with a RigidBody and a Collider component. In this case, we used a BoxCollider, an invisible box-shaped primitive that encloses the object. When a BoxCollider of an object came in contact with the collider of the player, the script initiated the vibration of the right Oculus Touch controller and registered a collision.

#### Questionnaire

A questionnaire was created to collect participants’ opinions on the overall experience and the 2 types of feedback. The questionnaire (Multimedia Appendix 3) included 8 items. Questions 1 to 3 were designed to investigate participants’ familiarity with VR, emotional reaction, and potential motion sickness felt during the experiment. Question 4 sought to understand participants’ personal perception of their navigation performance during the 4 experimental conditions. Question 5 asked for an explanation about their answer to question 4. Question 6 was designed to explore participants’ preference toward 1 specific condition. Question 7 required an explanation about that preference. Finally, the participants were asked to give an overall evaluation of the experience using a 5-point scale (not at all interesting, slightly interesting, moderately interesting, fairly interesting, and extremely interesting). The questionnaire was developed in a Google form, and it was accessible to participants by scanning a QR code. Participants filled out the questionnaire only after they completed all the 4 experimental conditions.

#### Data Processing

**Figure 5:**
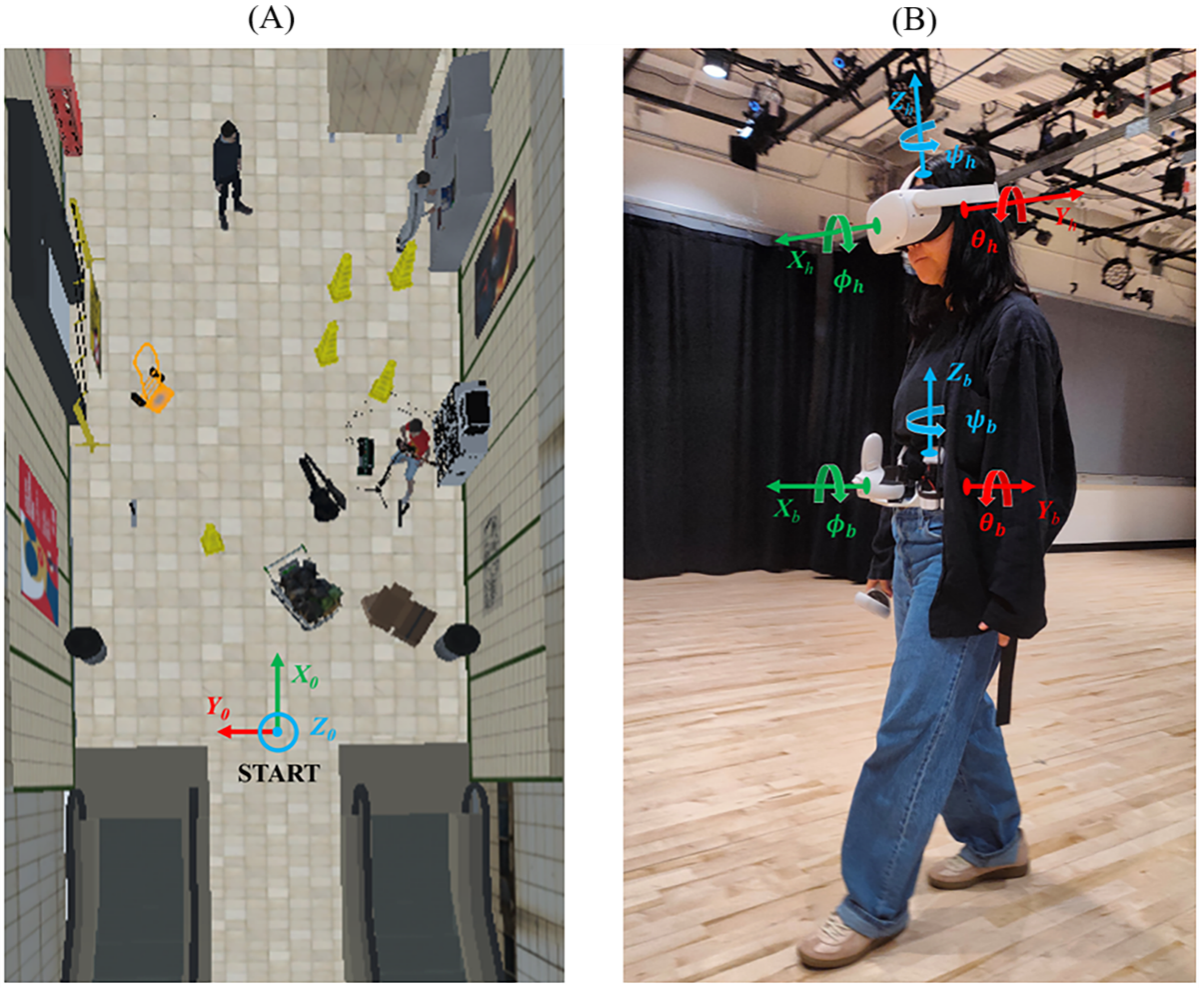
Coordinate systems used to define (A) body position (CS0; X0, Y0, and Z0) and (B) head (CSh; Xh, Yh, and Zh) and body (CSb; Xb, Yb, and Zb) orientation.

The data processing was performed in MATLAB (MathWorks, version 2021b). The body position was defined in a coordinate system CS0 whose origin was set at the experiment starting position, as shown in Figure 5A. The x- and y-axes were oriented along the main dimensions of the room, while the z-axis was aligned with the direction of gravity. Euler angles (*ψ_b_*, *θ_b_*, *ϕ_b_*) were used to describe the orientation of the trunk, and Euler angles (*ψ_h_*, *θ_h_*, *ϕ_h_*) were used to describe the spatial orientation of the head; coordinate systems are shown in Figure 5B. Raw data of the Euler angles and body position were smoothed using a quadratic regression method over a window of 20 samples to minimize noise from the measured data.

#### Trajectory Length and Smoothness

We computed participants’ trajectory length and smoothness. The trajectory length of each participant was calculated as follows:



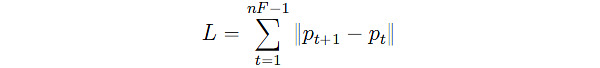



where *nF* is the number of frames, *p_t_* = [X*_0,t_*,Y*_0,t_*] is the body position in 2 dimensions at time step t, and ||・|| is the Euclidean norm.

Smoothness was estimated through the spectral arc length (SPARC) [61] and computed as follows. First, we performed a numeric derivative on the speed profile *v*. Then, we computed the fast Fourier transform on the speed to obtain the spectrum magnitude *V*(*f*) as a function of the frequency *f*, which we normalized with respect to its maximum to obtain.



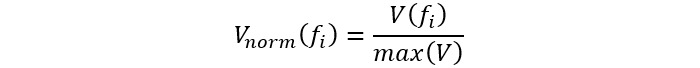



where *f_i_* is the *i-th* frequency component of the spectrum.

We determined the cut-off frequency *f_c_* as the maximum frequency where the spectral magnitude is above a threshold *V* and below a maximum frequency limit *f_max_*,

*f_c_* = {*f_i_* < *f_max_*, *V_norm_*(*f_i_*) > *V*}

Finally, we computed the SPARC,



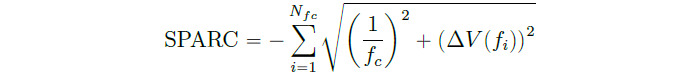



where *N_fc_* is the number of frequency components up to *f_c_* and *ΔV*(*f_i_*) is the difference in the normalized spectrum magnitude between adjacent frequency components, calculated as *ΔV*(*f_i_*) = *V_norm_*(*f_i+1_*) − *V_norm_*(*f_i_*). We set *V* = 0.05 and *f_max_* = 10 Hz. The SPARC is related to the frequency content of the velocity, and therefore, a smoother movement presents a higher value of SPARC.

#### Head and Body Motion Entropy

To evaluate how each condition affected the user’s head motion, we performed an analysis of the variability of the pitch angle of the head *θ_h_* and the difference between the head yaw angle *ψ_h_* and body yaw angle *ψ_t_*, defined as *χ*.

The angle variability was calculated by computing Shannon entropy, defined as



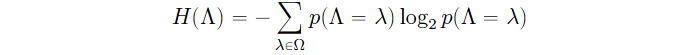



where *p*(∙) denotes probability and *λ* is a realization of *Λ* in the sample space of all the possible realizations *Ω*. The entropy *H*(*Λ*) is expressed in bits because a logarithm with base 2 was used. To compute the entropy for the aforementioned angles, we split the range of motion into single-degree intervals and computed the probability for each bin.

### Statistical Analysis

The statistical analysis was performed in RStudio (Posit PBC, version 2022.07.2). Specifically, the function *kolmogorov_test* of the *nortest* package (version 1.0-4) was used to perform the normality test on residuals. The function *lmer* of the *lmerTest* package (version 3.1-3) and the function *anova* of the *rstatix* (version 0.7.0) were used to conduct the 2-way repeated measures ANOVA. The function *rank* of the car package (version 3.1-2) was used for the rank transformation. The function *Scheirer-Ray-Hare* of the package 2.4.35 was used to conduct the Scheirer-Ray-Hare test. The graphical representations of the statistical analysis shown in the interaction plots were computed using the function *ggplot* of the *ggplot2* package (version 0.4.0).

Before the execution of the statistical analysis, we used the Kolmogorov-Smirnov test to evaluate the normality of residuals derived from our linear model. For each performance metric, we conducted normality tests across various experimental conditions, encompassing scenarios with no feedback, haptic feedback only, audio feedback only, and both feedback modalities. For the time taken to complete the task, trajectory length, entropy of the pitch angle, and difference between the yaw angle of the head and the yaw angle of the body, we found evidence to reject the null hypothesis that the data do not follow a normal distribution. However, for the number of collisions and trajectory smoothness, the test did not provide sufficient evidence to reject the null hypothesis. On the basis of these findings, we rank-transformed the trajectory smoothness and verified the normality of the residuals, akin to the other continuous metrics mentioned in the Metrics subsection, and chose an alternative test, Scheirer-Ray-Hare, for the specific treatment of the number of collisions, the only discrete metric of our study (whose residuals from a standard ANOVA would not satisfy the normality assumption).

To study the individual and synergistic effects of haptic and audio feedback on participants’ navigation performance (RQ1), we performed a 2-way repeated measures ANOVA on the following metrics: (1) time taken by each participant to complete the task across all conditions; (2) trajectory length, L, of each participant across all conditions; and (3) rank-transformed trajectory smoothness, SPARC, of each participant across all conditions.

We performed a Scheirer-Ray-Hare test on the number of collisions of each participant while performing the task across all conditions.

To address the individual and synergistic effects of the haptic and audio feedback on participants’ movement behavior (RQ2), we performed a 2-way repeated measures ANOVA on the following metrics: (1) entropy of the pitch angle of the head, *H*(*θ_h_*), of each participant across all conditions; and (2) entropy of the difference between the yaw angle of the head and yaw angle of the body, *H*(*χ*), of each participant across all conditions.

Finally, to gather participants’ opinion regarding their overall experience and their perceptions of the 2 types of feedback used across the 4 experimental conditions (RQ3), we conducted a descriptive statistical analysis of their answers to the questionnaire.

Before the statistical analysis, we identified outliers in the datasets. Out of 288 observations, the analysis revealed the presence of 6 (2.1%) outliers in the completion time dataset, 92 (31.9%) outliers in the number of collisions dataset, 25 (8.7%) outliers in the trajectory length dataset, 19 (6.6%) outliers in the SPARC dataset, 1 (0.4%) outlier in the entropy of pitch angle dataset, and 5 (1.7%) outliers in the dataset of the entropy of the difference between the yaw angle of the head and body yaw angle. The presence of outliers is ascribed to instances in which participants may have encountered challenges in comprehending the functioning of the devices or may not have paid attention to 1 or both feedback types. We removed all the outliers from the analysis*.*

### Ethical Considerations

Before starting the experiment, all participants signed an informed consent form in accordance with procedures approved by the Institutional Review Board at New York University (IRB-FY2023-7774). Participants were also told that they could take breaks between each condition and withdraw from the study at any time. All data collected during the study are nonidentifiable, ensuring participants' privacy and confidentiality. Furthermore, participants did not receive any compensation for their participation in the experiment.

## Results

**Figure 6:**
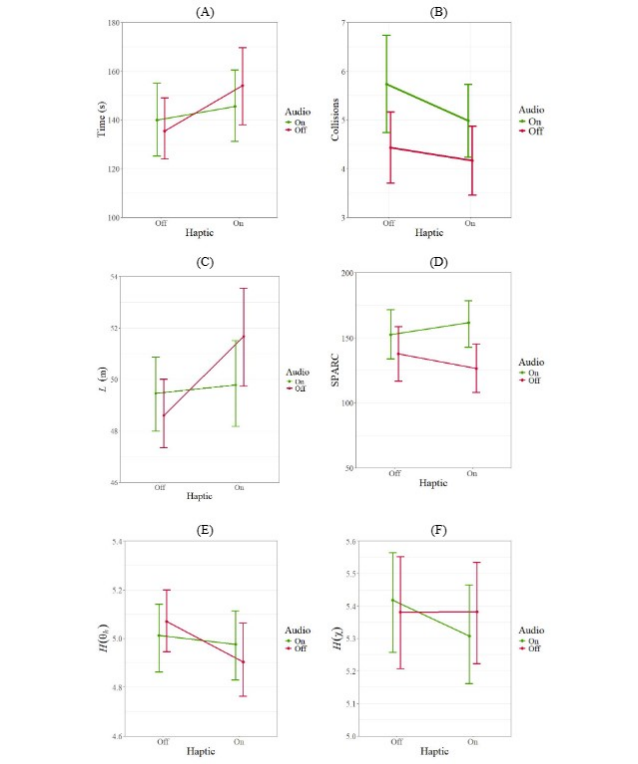
Interaction plots showing the individual and synergistic effect of the haptic and audio feedback on participants’ navigation performance: (A) time to complete the task, (B) number of collisions, (C) trajectory length (L), and (D) trajectory smoothness (spectral arc length; SPARC). Interaction plots showing the individual and synergistic effect of the haptic and audio feedback on participants’ movement behavior: (E) entropy of the head, H(θh), and (F) entropy of the difference between the yaw angle of the head and the yaw angle of the body, H(χ).

Experimental results in terms of mean and SE of the mean for individual and synergistic effects of haptic and audio feedback are reported in Figure 6. To determine the effectiveness of each feedback as well as their interaction, we conducted a 2-way repeated measures ANOVA on continuous metrics and a Scheirer-Ray-Hare test on discrete ones.

### Navigation Performance

#### Number of Collisions and Completion Time

Experimental results on completion time and number of collisions are reported in Figure 6A and Figure 6B, respectively. The haptic feedback through the belt was conducive to an increase in the completion time of the task (*F*_1,207.5_=4.7962; *P*=.03) and a decrease in the number of collisions (test statistic from the Scheirer-Ray-Hare test, H=3.8285; *P*=.05). The audio feedback, instead, was not found to modulate the completion time and number of collisions; neither did we find a main effect of the audio feedback (completion time: *F*_1,207.5_=0.1467; *P*=.70 and collisions: H=0.6110; *P*=.43), nor did we observe a significant interaction between the audio and haptic feedback (completion time: *F*_1,207.5_=1.7725; *P*=.18 and number of collisions: H=0.8518; *P*=.35).

#### Trajectory Length and Smoothness

Experimental results on L and SPARC are reported in Figure 6C and Figure 6D, respectively. The haptic feedback through the belt was linked to a notable increase in trajectory length (*F*_1,188.12_=7.3482; *P*=.007), though it did not yield a significant variation of the trajectory smoothness (*F*_1,213_=0.0127; *P*=.91). In contrast, audio feedback yielded a significant enhancement in the trajectory smoothness (*F*_1,213_=7.6342; *P*=.006), but it did not influence the trajectory length (*F*_1,188.09_=0.2972; *P*=.58). A significant interaction between haptic and audio feedback was observed with respect to the trajectory length (*F*_1,186.73_=4.20092; *P*=.04) but not with respect to the trajectory smoothness (*F*_1,213_=1.2684; *P*=.26).

### Movement Behavior

Experimental results on *H*(*θ_h_*) and *H*(*χ*) are reported in Figure 6E and Figure 6F, respectively. The haptic feedback through the belt resulted in a reduction of the entropy of the pitch angle of the head (*H*(*θ_h_*): *F*_1,208.54_=6.1273; *P*=.02), but it did not yield a significant variation in the entropy of the difference between the yaw angle of the head and the yaw angle of the body (*H*(*χ*): *F*_1,210.93_=1.5553; *P*=.21). Audio feedback was not found to influence either the entropy of the pitch angle of the head or the entropy of the difference between the yaw angle of the head and the yaw angle of the body (*H*(*θ_h_*): *F*_1,209.10_=0.0356; *P*=.85 and *H*(*χ*): *F*_1,210.93_=0.1791; *P*=.67). No significant interaction was observed between audio and haptic feedback *H*(*θ_h_*): *F*_1,208.54_=1.9633; *P*=.16 and *H*(*χ*): *F*_1,210.93_=1.0517; *P*=.31).

### Perception

From the analysis of the questionnaires, we found that 63% (45/72) of the participants had previous experience with VR, 76% (55/72) felt engaged while performing the experiment, and only 6% (4/72) experienced nausea or motion sickness while performing the experiment. We discovered that 50% (36/72) of the participants thought that their navigation performance, in terms of completion time and collision number, was better in the condition where they received both the haptic and audio feedback. In total, 24% (17/72) of the participants thought their navigation performance was better in the condition where they received only the audio feedback, and 14% (10/72) of the participants thought their navigation performance was better in the condition where they received only the haptic feedback.

We found that 40% (29/72) of the participants preferred the condition where they received both the haptic and audio feedback, 32% (23/72) of the participants favored the condition where they received only the audio feedback, and 18% (13/72) of the participants favored the condition where they received only the haptic feedback. Finally, 58% (42/72) of the participants evaluated the overall experiment as extremely interesting, and 39% (28/72) of the participants evaluated the overall experiment as fairly interesting.

## Discussion

### Context and Significance

VI refers to a condition where an individual experiences limited vision that cannot be fully corrected by glasses, contact lenses, or medical interventions. Persons with VI often encounter significant mobility issues that may affect their confidence in engaging with their surroundings, hindering social interactions and community involvement. Ongoing advancements in ETAs continue to contribute to the increased autonomy and improved mobility of individuals with VIs, highlighting the potential of technology to positively impact the lives of those facing mobility challenges. These devices leverage technology to assist users in navigating their surroundings more effectively. Common characteristics of ETAs include the use of sensors, GPS technology, and auditory or tactile feedback systems to detect obstacles and provide users with real-time feedback about their environment or to help users with route planning and destination guidance.

In this study, we introduced a multisensory AT system based on haptic and audio feedback for obstacle avoidance. We tested our system in a VR environment resembling a complex urban environment. VR offers the possibility to design highly realistic and easily customizable environments where ATs can be tested and refined under various experimental conditions while avoiding potential risks of the real world. In addition, rendering and postprocessing tools available in VR enable an accurate simulation of various forms of VI at different stages of progress. While we recognize that studying healthy participants with simulated VI does not fully replicate real-life scenarios of organic VI individuals, it is a critical first step in developing ATs. Using healthy participants in early technology phases allows us to test and refine ATs without causing stress for actual VI individuals, who may prefer later-stage trials. Recognizing the importance of inclusivity in participant selection, we intend to expand our research to include a broader range of persons with blindness or various experiences of VI, including those with acquired or congenital eye pathologies, to ensure the clinical relevance of our findings.

We extended our previous work on the use of VR for testing, refining, and training with ETAs [42]. We proposed a multisensory system where haptic feedback is provided by an upgraded version of our in-house built haptic feedback device [37-39], complemented by audio feedback that is provided by a VR headset. The system was evaluated through an experiment where 72 healthy participants performed an obstacle avoidance task in a virtual subway station while experiencing the simulation of VI symptoms at an advanced severity stage. The virtual environment was designed to align with the dimensions of the physical environment where the experiment took place. During the experiment, participants were asked to walk in the VR environment trying to avoid obstacles that were presented along their path. Each participant performed the experiment 4 times under different conditions (with haptic feedback only, with audio feedback only, with both haptic and audio feedback, and without any feedback). Depending on the experimental condition, participants received vibrotactile feedback on the abdomen through the belt and audio feedback, consisting of a beep sound, from the VR headset that indicated the presence of obstacles along their path.

Through this experiment, we investigated the impact of our multisensory, AT-integrated VR system on participants’ mobility performance and movement behavior. Specifically, we evaluated how the individual and synergistic use of the 2 types of feedback affected the navigation performance (RQ1), movement behavior (RQ2), and perception (RQ3) of participants across experimental conditions. We performed a 2-way repeated measures ANOVA on task completion time, number of collisions, trajectory length and smoothness (RQ1), the entropy of the pitch angle, and the entropy of the difference between the yaw angle of the head and the yaw angle of the body (RQ2). Finally, we conducted a descriptive statistical analysis of their answers to the questionnaire (RQ3).

### Principal Findings

#### Navigation Performance and Movement Behavior

Our investigation of the efficacy of the haptic feedback device indicated notable improvements in participants’ navigation performance, specifically in reducing the number of collisions. However, these positive effects did not extend to task completion time, trajectory length, or trajectory smoothness. Contrary to our expectations, the introduction of the haptic feedback device led to a significant increase in task completion time. Participants exhibited hesitancy in their walking behavior when relying only on the haptic feedback device, as evidenced by observable delays in reacting to stimuli. Such an outcome diverged from our earlier work [42], where the haptic feedback device was found to reduce task completion time. This disparity can be attributed to the increased difficulty and duration of the obstacle avoidance task in this study as well as the distinct walking modality used. In this experiment, participants navigated a dynamic and complex urban environment, whereas in our previous study, they traversed a simpler and smaller outdoor environment using a controller. The prolonged task completion time resulted in longer trajectories in response to haptic feedback.

The spatial resolution of the haptic feedback device played a crucial role in these findings, as the detailed environmental information prompted participants to navigate cautiously, resulting in intricate trajectories. Examining participants’ movement behavior, we observed a significant reduction in the entropy of the pitch angle of the head due to haptic feedback. Just as participants moved more smoothly in the environment, they also maintained a more constant and less variable head orientation. However, the device did not affect the entropy of the difference between the yaw angle of the head and the yaw angle of the body. This result may be attributed to the spatial information provided by the haptic feedback device, which guided users based on their body orientation and prompted them to reduce their vertical head movements.

We registered an effect of audio feedback on participants’ navigation performance with respect to trajectory smoothness. Using audio feedback, participants were likely to favor straight paths, as seen from reduced instances of halted movement and a reduced tendency to course-correct during navigation. Moreover, the design of the audio feedback system, which alerted users to obstacles in their line of sight, may have facilitated the exploration of the environment with just the movement of their head. We did not register a variation in the number of collisions, likely due to the modality used by the audio feedback device for obstacle detection. In contrast to the haptic feedback device, which detected obstacles using 10 vibrating actuators on the user’s abdomen, the audio feedback device signaled the presence of obstacles in the user’s line of sight through a distinctive beep sound emitted by the VR headset. The lower spatial resolution of the audio feedback device may have been less effective in aiding users to avoid obstacles compared to the haptic feedback.

While not effective in reducing the trajectory length alone, audio feedback had a positive effect on haptic feedback in the form of a significant interaction between the 2 modalities. In fact, the increase in trajectory length due to haptic feedback alone is mitigated by the concurrent use of audio feedback, thereby suggesting that participants were able to leverage both information cues and make informed decisions as they negotiated haptic versus audio cues. However, a positive role of combined feedback was not observed for all metrics, likely due to the increased cognitive load resulting from the use of both feedbacks. It is tenable that the delivery of multiple feedback cues poses some difficulties in terms of assimilation and requires a learning curve for users to adapt to new approaches. In principle, this may be mitigated by increasing the training time for users to become more proficient with combined feedback. Overall, the combined use of audio and haptic feedback enhances safety by facilitating informed decision-making, and it contributes to travel efficiency by addressing trajectory length and smoothness. This underscores the potential of blending feedback modalities to optimize both safety and travel efficiency.

#### Participants’ Feedback

The results of the questionnaire offer valuable insights into participants’ experiences and preferences during the experiment. The high engagement reported by 76% (55/72) of the participants suggests that the multisensory feedback, comprising both haptic and audio cues, contributed to an immersive and captivating experience. Notably, only a minimal percentage of participants (4/72, 6%) experienced nausea or motion sickness, indicating that the implemented feedback modalities were well tolerated. Participants’ perceptions of navigation performance revealed a preference for the combined haptic and audio feedback condition, with 50% (36/72) of them believing that it enhanced their performance. Participants emphasized that haptic and audio cues offer distinct information. Many participants note that having both types of feedback provides a more complete and nuanced understanding of their surroundings, aiding in better decision-making and spatial awareness. Finally, others found the combination more intuitive, with haptic feedback offering directional cues and audio feedback providing information on the proximity of obstacles. The preference for both modalities suggests that, when used together, they complement each other, addressing potential limitations or confusion that might arise when using either haptic or audio feedback alone. Interestingly, the preferred condition did not always align with perceived performance, highlighting the complexity of user preferences. Finally, most of the participants (42/72, 58%) found the overall experiment extremely interesting, emphasizing the potential of multisensory, AT-integrated VR systems in maintaining user engagement. These findings underscore the importance of considering user preferences and experiences when developing and refining multisensory ATs, ensuring that future iterations are tailored to meet the needs of individuals with visual impairments.

### Limitations

Our study is not free of limitations with respect to the wearable design, VR environment, and experimental approach. Specifically, we identified the following 5 main limitations.

First, we used only 1 type of audio feedback. We cannot exclude the possibility that other forms of audio feedback may have different effects on our haptic feedback system. We chose this particular design for the audio feedback after pilot trials because it offered a straightforward and intuitive means for users to access environmental information, aiding them in obstacle avoidance. In the future, we plan to design experiments that will involve the evaluation of auditory cues individually and in combination to assess their impact on participants’ task performance and overall user experience. Specifically, participants will be immersed in virtual environments simulating crowded urban settings and real-world challenges, including navigating through busy intersections, crossing streets safely, and locating specific points of interest within the urban environment. These tasks will provide valuable insights into how different types of audio feedback can influence participants’ navigation strategies, decision-making processes, and overall spatial awareness in crowded urban environments.

The second limitation pertains to simulating only the most common symptoms of VI. While glaucoma is a prevalent eye pathology and our methodology can be readily expanded to other eye pathologies, we acknowledge the need for future research to tackle a wider range of end users. Specifically, we anticipate the development of new systems, incorporating varied forms of audio and haptic feedbacks and tested in diverse conditions and with individuals experiencing different eye pathologies. Testing our system on various types and forms of VI could provide more robust evidence, demonstrating broader applicability to a diverse range of users.

The third limitation arises from the fact that, in the real world, individuals would exercise caution in avoiding obstacles to prevent injury. This instinctive behavior may not be fully present in VR environments, where collisions do not result in any negative consequences. As a result, participants might prioritize completing the task quickly over minimizing the number of collisions. One potential strategy to mitigate this issue involves introducing incentives, such as rewards, or placing cardboard obstacles in the environment to encourage participants to focus more on avoiding obstacles rather than completing the experiment quickly.

A fourth limitation is related to the number and placement of actuators on the ETA used in the research. The current configuration of actuators was determined based on a practical balance between providing enough information and avoiding overwhelming users with excessive tactile stimuli. However, the optimal arrangement and quantity of actuators may vary among individuals, as sensory preferences and sensitivities can differ widely. Recognizing this limitation, we acknowledge the necessity of future investigations that explore alternative configurations of actuators on the ETA. Our upcoming research plans include testing different numbers and arrangements of actuators to identify an optimal solution that caters to the diverse sensory needs of users with VI. This iterative approach aims to enhance the user experience and effectiveness of the multisensory, AT-integrated VR system, ensuring its adaptability and usability across a broader spectrum of individuals with varying preferences and sensitivities.

Finally, it is essential to acknowledge that our study only involved healthy participants with simulated VI. Such an experimental choice limits the direct applicability of our findings to the broader community of individuals with VI and the practical implication of the proposed ETA. At the same time, our research provides valuable insights into the use of VR in research disability and serves as an important preliminary step in the development of ATs tailored to address the specific needs of individuals with VI. Recognizing the significance of simulating real-world challenges within the VR environment for effective rehabilitation interventions, we will broaden our research scope to encompass diverse neurological conditions. In our upcoming studies, we plan to include individuals with balance and neurological issues to further explore the applicability of our multisensory AT solutions in rehabilitation settings. Doing so will undoubtedly enhance the clinical relevance and generalizability of our findings, aligning with our overarching goal of developing and validating tailored interventions for various clinical populations.

### Conclusions

AT for persons with VI plays a pivotal role in enhancing their sensory perception and spatial awareness. These devices often integrate a combination of auditory, haptic, and visual cues to provide comprehensive information about the surrounding environment. However, most devices are designed without a user-centered focus, often featuring complexities beyond consumer necessity [62-64]. Research needs to hone methodologies that better support consumer-oriented and user-centered devices as well as test and evaluate them in realistic scenarios while limiting safety issues and concerns for persons with VI. This holistic approach aims to bridge the gap between theoretical advancements and practical applications, ultimately enhancing the usability and impact of ETAs on the lives of individuals with VI. Our multisensory, AT-integrated VR system is a first step in this direction that may enhance the user’s ability to interpret and interact with their surroundings. Our synergistic approach facilitates safer mobility with improved travel efficiency and opens avenues for innovative applications in areas such as education, training, and rehabilitation for persons with VI.

In our forthcoming research, we aim to enhance and evaluate our multisensory AT-integrated VR system for persons with VI. This endeavor will be guided by a comprehensive methodology that encompasses various domains of knowledge and caters to the diverse needs of the target population. Our design process will take into account the wide spectrum of VI, which ranges from low vision to total blindness, considering the varying degrees of VI and potential additional impairments such as hearing loss or peripheral neuropathy. In addition, we will acknowledge the diversity within the VI population in terms of visual experience, spanning from congenital blindness to acquired blindness later in life, which can significantly influence their interaction with ATs [65]. Central to our approach is understanding user preferences, technological familiarity, and motivation, as these factors are pivotal for the acceptance and effectiveness of AT devices [65]. The experimental phase will include a cohort of healthy participants and individuals with VI. The VR setting will be equivalent for both groups to ensure consistency and comparability of results. By comparing the experimental outcomes between the 2 groups, we aim to pinpoint limitations associated with experiments performed solely on healthy participants, particularly those related to sensory compensation. In addition, this comparison will help identify the behavioral traits that are preserved when experimenting with healthy participants, providing valuable insights for the development and optimization of our multisensory AT in real-world clinical settings.

## Appendix

Multimedia Appendix 1 A video showcasing the virtual reality environment used for evaluating the multisensory assistive technology outlined in this study, providing the perspective of an individual with normal vision.

Multimedia Appendix 2 A video displaying the virtual reality environment used to assess the multisensory assistive technology outlined in this study, offering the perspective of an individual experiencing simulated symptoms of visual impairment.

Multimedia Appendix 3 The questionnaire administered to participants through a Google form at the conclusion of the experiment.

## Abbreviations

AT: assistive technology
ETA: electronic travel aid
RQ: research question
SPARC: spectral arch length
VI: visual impairment
VR: virtual reality
