# Supplementary material for: Navigation Training for Persons With Visual Disability Through Multisensory Assistive Technology: Mixed Methods Experimental Study
Source: JMIR Rehabil Assist Technol. 2024 Nov 18;11:e55776. doi: 10.2196/55776 (PMC11612587; doi:10.2196/55776)
Supplement: Multimedia Appendix 3 [file rehab_v11i1e55776_app3.pdf]

Post- Experiment Questionnaire

1. Email *

____

2. Do you have any previous experience with VR?

Mark only one oval.

- [ ] yes
- [ ] no

3. How did you feel while performing the task?

Mark only one oval.

- [ ] engaged
- [ ] bored
- [ ] indiffirent
- [ ] overwhelmed
- [ ] Other: ______________________________

4. Did you experience nausea or motion sickness during the task?

Mark only one oval.

- [ ] yes
- [ ] no

5. Under which of the four conditions do you think your navigation performance, in terms of completion time and number of collisions, have been better?

Mark only one oval.

- Only haptic
- Only audio
- Both haptic and audio
- No haptic and no audio

6. Based on you previous answer, why do you think you performed better in that specific condition?

____

7. Is there a particular condition you preferred the most?

Mark only one oval.

- Only haptic
- Only audio
- Both haptic and audio
- No haptic and no audio

8. Based on you previous answer, why do you prefer that specific condition ?

____

9. In a scale from 1 to 5, how do you evaluate this experience?

Mark only one oval.

	1	2	3	4	5	
Not	( )	( )	( )	( )	( )	Extremely interesting

This content is neither created nor endorsed by Google.

Google Forms
